# Leveraging deep learning for prognostic stratification of hepatocellular carcinoma using histopathological images

**DOI:** 10.3389/fonc.2026.1849274

**Published:** 2026-09-28

**Authors:** Dengbin Wu, Jiaoyue Zhang, Mian Shi, Baoku Xu, Shijian Fan, Wei Zhang, Fanli Wang

**Affiliations:** 1 Ansteel Group Hospital, Anshan, China; 2 Anshan Center for Disease Control and Prevention, Anshan, China

**Keywords:** AI deep learning, hepatocellular carcinoma, histologically stained slide, histopathological biomarkers, prognostic prediction model, prognostic stratification

## Abstract

**Background:**

Hepatocellular carcinoma (HCC) is one of the most common and lethal malignancies, whose prognostic prediction is particularly challenging. Histopathological biomarkers are critical for cancer prognosis assessment. Advancements in artificial intelligence (AI) have made it possible to automate the classification and detection of information from whole-slide pathology images using deep learning. This study aims to utilize AI deep learning to analyze histopathological slides of HCC patients for prognostic stratification, thereby assisting in the development of clinical treatment plans.

**Methods:**

The Otsu thresholding method was employed to automatically segment whole-slide images (WSIs) from two HCC datasets, The Cancer Genome Atlas (TCGA) and HCC slides from Ansteel General Hospital. The pathological features of WSIs, extracted by the pretrained CONCH model were utilized as model inputs. Model performance was assessed using the area under the receiver operating characteristic curve and additional metrics. Then Attention Heatmap Visualization was employed to analyze interpretability. The Kolmogorov-Smirnov curve was utilized to stratify patients into distinct risk groups. Survival analysis was performed to evaluate differences in overall survival between distinct risk groups. Univariate and multivariate Cox regression analyses were used to identify key clinical indicators and pathological features as significant predictors. The riskRegression package generated calibration curves comparing predicted versus observed survival outcomes. Calibration curves were quantify the deviation between model-predicted probabilities and actual observed outcomes.

**Results:**

A prognostic prediction model was developed using the CLAM self-attention framework based on TCGA dataset, whose generalization capability was verified by histologically stained slide data from a proprietary dataset. The results showed that the model could achieve better migration. Mortality rates demonstrated a progressive increase across ascending risk groups. The risk stratification system possesses potential clinical utility for guiding treatment selection. Forest plots generated demonstrated that the risk stratification system functions as an independent prognostic predictor for HCC. Calibration curve analysis confirmed the predictive accuracy of the model, and this finding from Decision Curve Analysis underscores its superior utility in guiding clinical decision-making.

**Conclusion:**

This study developed a prognostic prediction model, whose risk stratification could be an independent prognostic predictor for HCC and a guide for therapeutic decision-making.

## 1 Introduction

Hepatocellular carcinoma (HCC) ranks among the most prevalent and fatal malignancies, with 5-year survival rates exhibiting significant variability across different populations and averaging below 20% (1, 2). The pronounced heterogeneity and intricate etiological factors associated with HCC complicate accurate prognostic predictions (3). Additionally, the scarcity of therapeutic options for HCC highlights the urgent requirement for dependable prognostic tools to enable precision treatment (4). Histopathological biomarkers are pivotal in cancer diagnosis, prognostic evaluation, and treatment planning. Recent advancements in artificial intelligence (AI), particularly in deep learning, have facilitated the automated extraction, classification, and detection of histopathological features from whole-slide images (5–9). The swift progress of AI has also resulted in its increasingly widespread application in medicine, especially in medical imaging (10). By offering an objective and quantitative approach to image analysis, AI can diminish reliance on subjective visual assessments and potentially enhance diagnostic accuracy, thereby advancing precision medicine (11). This study aimed to employ deep learning on histopathological whole-slide images from HCC patients to perform prognostic stratification, thereby supporting individualized clinical decision-making.

## 2 Materials and dataset

### 2.1 Data acquisition

This study used two HCC datasets for analysis. The first dataset was sourced from The Cancer Genome Atlas (TCGA), a comprehensive public repository of multi-omics cancer data, and included 365 patients with 381 hematoxylin and eosin (H&E)-stained HCC slides (12). The second dataset consisted of 111 patients with 124 HCC slides, which were obtained from Ansteel General Hospital.

### 2.2 Dataset preparation

The Otsu thresholding method was applied in this study to automatically segment whole-slide images (WSIs) and identify tissue regions (13). The identified tissue regions were subsequently divided into tiles measuring 256 × 256 pixels for further processing. The total number of tiles (N) varied according to the dimensions of each slide (7).

The Contrastive learning from Captions for Histopathology (CONCH) model is based on the advanced vision-language foundation model CoCa, incorporating an image encoder, a text encoder, and a multimodal fusion decoder (14). The CONCH model underwent pretraining with contrastive alignment objectives using a diverse set of histopathological images, biomedical texts, and over 1.17 million image-caption pairs (15). Consequently, the pretrained CONCH model can address various downstream tasks, such as histopathological image classification and segmentation, caption generation, text-to-image retrieval, and image-to-text retrieval, without requiring additional task-specific supervised fine-tuning. These features highlight its significant potential and extensive applicability in the field of computational pathology (15).

For each preprocessed 256 × 256-pixel image tile, a 512-dimensional feature representation was extracted using the pretrained CONCH model. This process generated an N × 512 feature matrix for each WSI. The slide-level representation was then obtained by averaging the feature vectors of all tiles, resulting in a single 512-dimensional vector for each WSI.

### 2.3 Prognostic prediction modeling based on dataset

After dividing the WSIs into fixed-size patches, 512-dimensional pathological feature vectors, extracted using the pretrained CONCH v1 model, served as inputs to the CLAM model. Within the multiple instance learning (MIL) framework, each WSI was considered a bag containing multiple patches (instances), with a slide-level label assigned to each bag for model training. The Clustering-constrained Attention Multiple Instance Learning (CLAM) framework specifically used an attention mechanism to assess the contribution of each patch to the final slide-level prediction. The model generated an attention score for each patch, indicating its relative importance in forming class-specific slide-level representations. These attention scores were subsequently used as weights in an attention-based pooling operation to consolidate patch-level features into a slide-level embedding, which was then used for prognostic prediction (16).

Model training was conducted utilizing the attention-based Multiple Instance Learning (MIL) framework as implemented in CLAM. The attention dimension was configured to 128, with a dropout rate of 0.25. The BCEWithLogitsLoss function was used, incorporating a positive class weight determined by the death-to-survival ratio within each training dataset. The Adam optimizer was applied with a learning rate of 1 × 10^-4^ and a weight decay of 1 × 10^-5^. The batch size at the bag level was set to 1, and model parameters were updated following gradient accumulation over eight patients. Training spanned a minimum of 12 epochs and a maximum of 40 epochs. Early stopping criteria were based on the Receiver Operating Characteristic Area Under the Curve (ROC-AUC) of the TCGA validation set, with a patience of 8 epochs and a minimum improvement threshold of 0.002. The model that achieved the highest validation ROC-AUC was selected. During training, up to 4,096 patches were randomly sampled from each patient, while up to 8,192 patches were sampled during validation and testing. A base random seed of 20260807 was utilized, and 10 independent training runs were executed using seeds ranging from 20260807 to 20260816. To enhance reproducibility, deterministic cuDNN operations were enabled, and cuDNN benchmarking was disabled.

Specifically, the TCGA cohort was stratified into training, validation, and internal test sets at a ratio of 7:1:2. Ten independent stratified data-splitting and model-training runs were performed (17–20). Stratification was used to preserve the class distribution across the training, validation, and internal test sets, thereby maintaining distributions comparable to that of the overall cohort.

### 2.4 Model validation and external generalization

Model performance was rigorously evaluated using the area under the receiver operating characteristic curve (AUC), together with additional performance metrics, including accuracy, recall, and F1-score (12).

To assess the model’s generalizability, an independent external validation cohort was employed, consisting of H&E-stained histopathological slides from our institution. This cohort comprised 47 slides from 42 deceased patients and 77 slides from 69 surviving patients. Notable heterogeneity was identified between the external cohort and the TCGA cohort, encompassing variations in sex distribution, disease severity, slide preparation procedures, and staining protocols. To address these domain shifts, 20% of the external cohort was utilized for model adaptation, while the remaining 80% was preserved as a held-out test set for independent performance evaluation. The model outputs were primarily applied for patient risk stratification and outcome discrimination.

### 2.5 Interpretability analysis via attention heatmap visualization

To evaluate the relative significance of various histopathological regions in model predictions, we calculated and preserved unnormalized attention scores for all extracted patches via the attention branch associated with the model-predicted class. These initial attention values were transformed into percentile-ranked scores and subsequently normalized to a 0–1 scale through min-max scaling, where a score of 1.0 signifies maximal attention (i.e., the highest contribution to the model prediction) and a score of 0 signifies minimal attention (21). The pathological evaluation of the attention heatmaps was independently conducted by two pathologists, who were blinded to both the clinical outcomes and the model predictions. Each pathologist assessed the spatial alignment between the regions of high attention and the relevant histomorphological features, such as tumor cellularity, necrosis, and stromal infiltration. Any disagreements were resolved through joint review and discussion to achieve consensus. The evaluation was qualitative, emphasizing the anatomical and morphological localization of model attention rather than employing quantitative scoring.

The normalized scores were subsequently mapped to a spectral RGB colormap and superimposed on their original spatial coordinates within whole-slide images (WSIs). This standardized visualization protocol enables intuitive identification of diagnostically salient regions:

High-attention zones (red hues) indicate histopathological features that disproportionately influence model decisions.

Low-attention zones (blue hues) indicate regions with negligible contributions to prediction.

### 2.6 Risk stratification based on Kolmogorov-Smirnov curve analysis

The Kolmogorov-Smirnov (K-S) curve was used to categorize patients into distinct risk groups. This nonparametric method, derived from the Kolmogorov-Smirnov test, evaluates classifier performance by measuring the maximum distance, known as the K-S statistic, between the cumulative distribution functions of predicted probabilities for the two outcome groups. A larger K-S statistic indicates improved discriminative performance of the classifier. The optimal classification threshold was identified at the point where the difference between the true positive rate (TPR) and false positive rate (FPR) was maximized. For prognostic stratification in hepatocellular carcinoma, the optimal cutoff for the risk score was determined using the scikit-learn package in Python (version 1.2.2). Patients were then classified into high_risk and low_risk groups based on this threshold.

### 2.7 Survival analysis for risk_group Stratification

Survival analysis was conducted to assess the differences in overall survival (OS) between high_risk and low_risk groups. OS was defined as the duration from the designated baseline time point to death from any cause. Patients who were still alive at the last follow-up were considered censored observations, with survival times calculated up to the date of their last follow-up.

The OS probabilities were estimated using the Kaplan–Meier (K–M) method, and survival curves were generated to visualize differences between the risk groups. Median OS and the corresponding 95% confidence intervals (CIs) were calculated when estimable. All survival analyses and visualizations were performed using the R packages survival (version 3.5-7) and survminer (version 0.4.9).

### 2.8 Univariate and multivariate Cox regression analyses

To assess the associations between the risk score, relevant clinical variables, and OS, a univariate Cox proportional hazards regression analysis was conducted. The analysis yielded hazard ratios (HRs), 95% CIs, and corresponding P values. Variables that demonstrated statistically significant associations in the univariate analysis (P < 0.05) were subsequently incorporated into a multivariate Cox regression model. This step aimed to ascertain whether the risk score persisted as an independent prognostic factor after adjusting for potential confounders.

### 2.9 Comparison of treatment strategies across different risk groups

As our institutional dataset contained detailed information on the treatment strategies received by each patient, we compared treatment patterns between the different risk groups. The major treatment modalities included immunotherapy, targeted therapy, radiotherapy, surgery, and transarterial chemoembolization (TACE). Treatment combinations and their intersections were visualized and analyzed using the UpSetR package in R.

### 2.10 Construction and validation of a prognostic nomogram

Utilizing significant predictors identified through univariate and multivariate Cox regression analyses, a prognostic nomogram was developed by integrating key clinical variables and pathological features with the rms package in R (version 6.7-0). This nomogram offers an individualized visual scoring system by allocating points to each predictor based on its contribution in the multivariable Cox model, thereby estimating the corresponding survival probabilities. Calibration curves were generated using the riskRegression package in R (version 2023.12.05) to evaluate the concordance between predicted and observed survival probabilities at 1, 3, and 5 years, with 95% CIs employed to describe the uncertainty of the calibration estimates.

### 2.11 Statistical analysis

Continuous variables were described using medians and interquartile ranges (IQRs), while categorical variables were expressed as frequencies and percentages. For between-group comparisons of continuous variables, the Mann–Whitney U test, Student’s t-test, or one-way analysis of variance (ANOVA) was employed, depending on the context. Categorical variables were compared using either the chi-square test or Fisher’s exact test, as appropriate. All statistical tests were conducted as two-sided, with a P value of less than 0.05 indicating statistical significance. Statistical analyses were executed using R (version 4.4.1) and Python (version 3.10.14).

## 3 Result

### 3.1 Model performance evaluation

The TCGA dataset was partitioned into training, validation, and internal test sets at a ratio of 7:1:2. A prognostic prediction model was developed using the CLAM self-attention framework. Model performance was evaluated using 10 independent iterations of stratified k-fold cross-validation, with the area under the receiver operating characteristic curve (AUC), accuracy, precision, recall, and F1-score used as evaluation metrics (**Table 1**). **Figure 1A** shows the receiver operating characteristic (ROC) curve for the optimal model in the training set, with an AUC of 0.836. **Figure 1B** shows its performance in the internal test set, with an AUC of 0.800, indicating robust predictive performance within the TCGA cohort.

**Table 1:** The model validation performance metrics based on training, validation, and internal test sets.

Model	Accuracy	F1-score	Precision	Recall	ROC-AUC
Traning set	0.757	0.598	0.705	0.519	0.836
Internal test set	0.760	0.609	0.700	0.538	0.800
External test set	0.645	0.569	0.527	0.617	0.704

**Figure 1:**
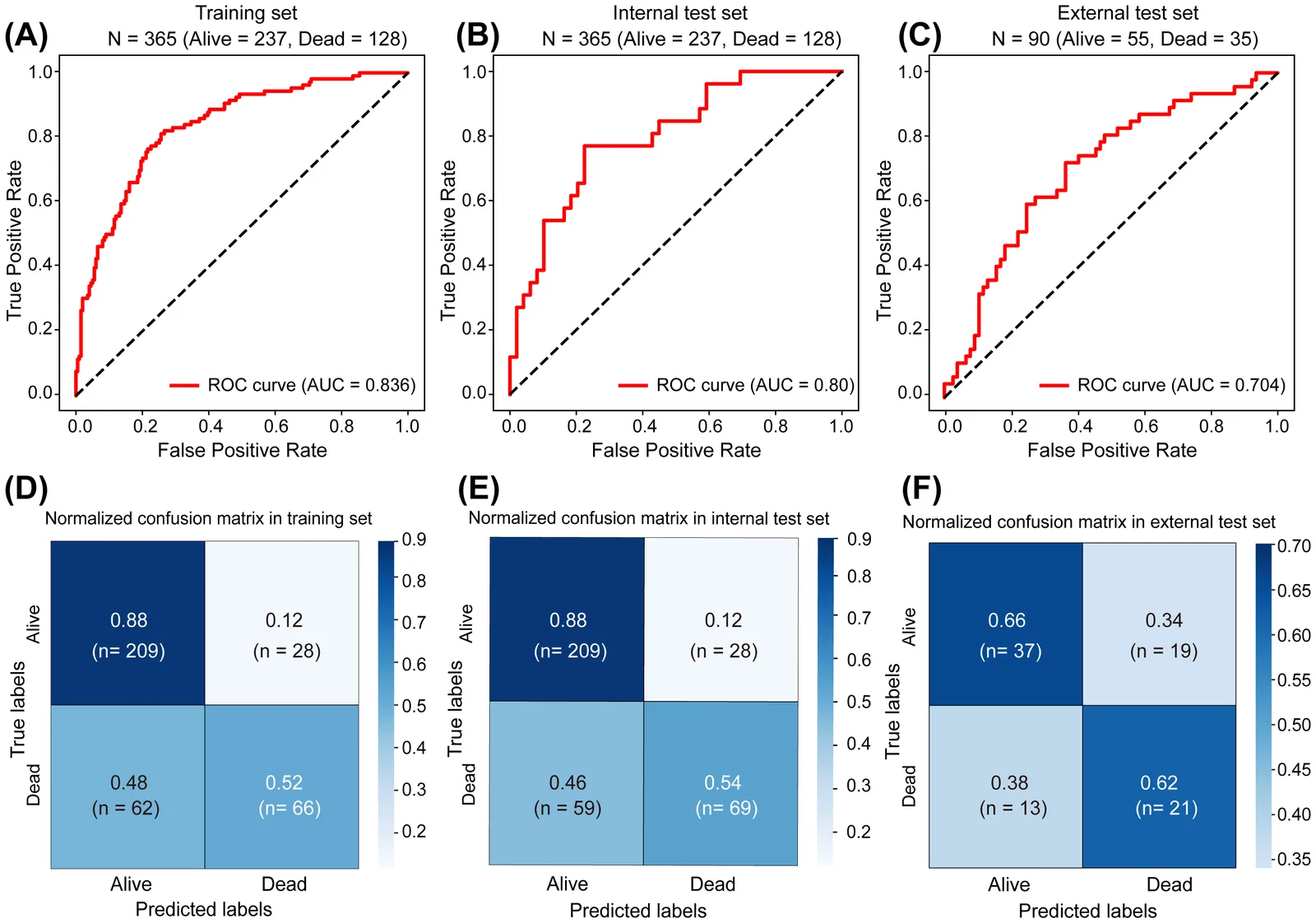
Development and validation of the prognostic prediction model based on the CLAM self-attention framework. The model was validated using 10 independent iterations of stratified k-fold cross-validation. Normalized confusion matrices were used to assess model performance across different datasets. **(A)** ROC curve of the optimal model in the TCGA training set. **(B)** Performance of the optimal model in the TCGA internal test set. **(C)** Performance of the model in the external test set from the proprietary dataset of Ansteel General Hospital. **(D)** Normalized confusion matrix of the optimal model in the training set. **(E)** Normalized confusion matrix of the internal test set. **(F)** Normalized confusion matrix of the external test set.

To evaluate the generalizability of the model, histologically stained slide data from a proprietary cohort were collected for external validation. The external cohort comprised 47 slides from 42 deceased patients and 77 slides from 69 surviving patients. To facilitate domain adaptation, 20% of the external cohort was used for model adjustment, while the remaining 80% was retained as an independent external test set. The model achieved an AUC of 0.704 in the external test set (**Figure 1C**), demonstrating acceptable predictive performance in an independent cohort. Furthermore, analysis of the normalized confusion matrices showed that the model achieved correct classification rates of 0.88, 0.88, and 0.66 for surviving patients in the training (**Figure 1D**), internal test (**Figure 1E**), and external test sets (**Figure 1F**), respectively.

Furthermore, to identify highly predictive regions, attention-score heatmaps were generated using the best-performing model. **Figures 2**, **3** shows representative whole-slide images (WSIs), their corresponding attention heatmaps, and high-attention patches from deceased and surviving patients.

**Figure 2:**
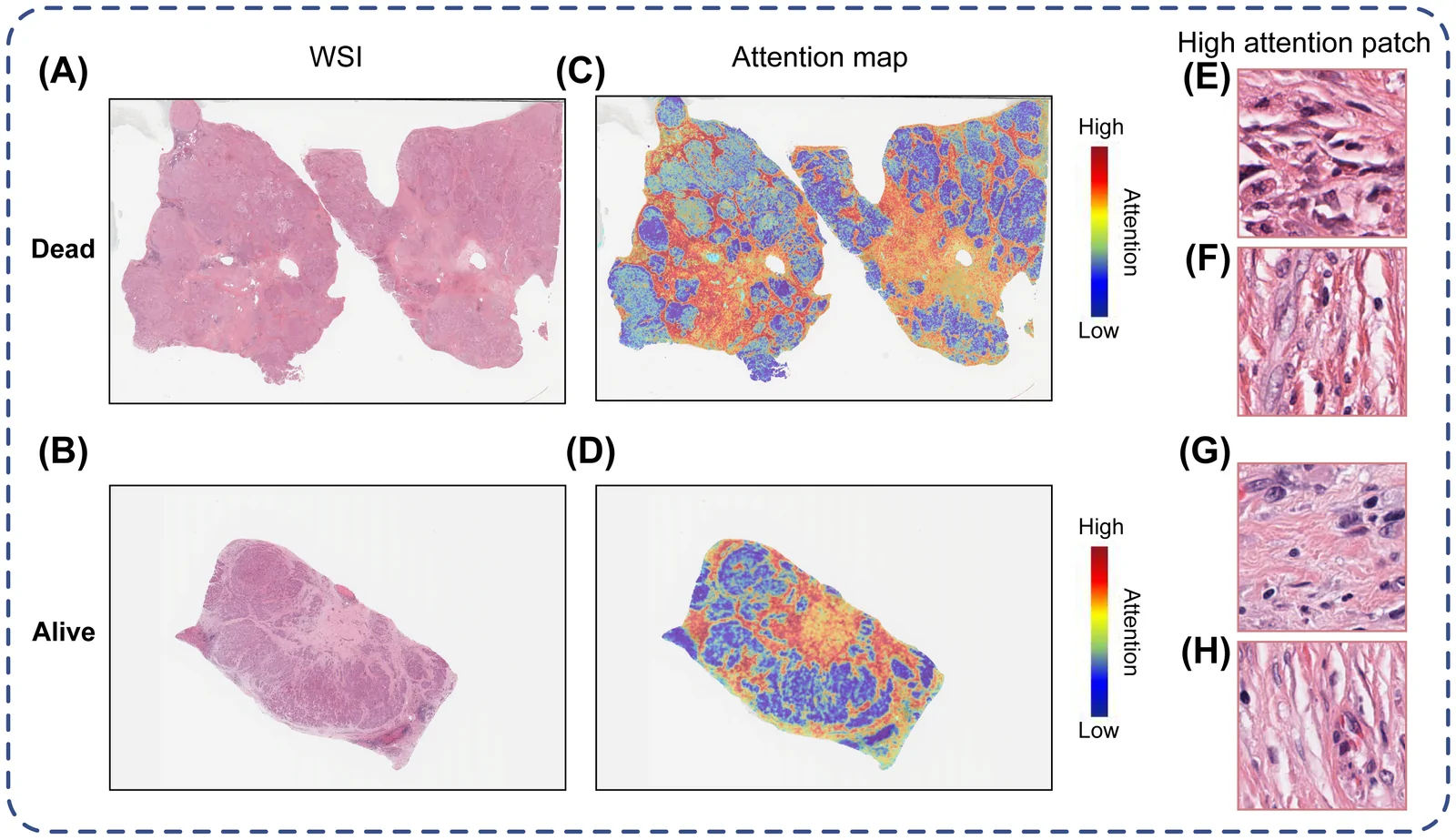
Attention score heatmaps. **(A)** Representative WSIs from a deceased patient. **(B)** Representative WSIs from a surviving patient. **(C)** The corresponding heatmap for **(A)**. **(D)** The corresponding heatmap for **(C)**. **(E)** Interstitial reaction and **(F)** immune infiltration. High-attention patch samples from a deceased patient. **(G)** Mitotic figure and **(H)** necrosis. High-attention patch samples from a surviving patient.

**Figure 3:**
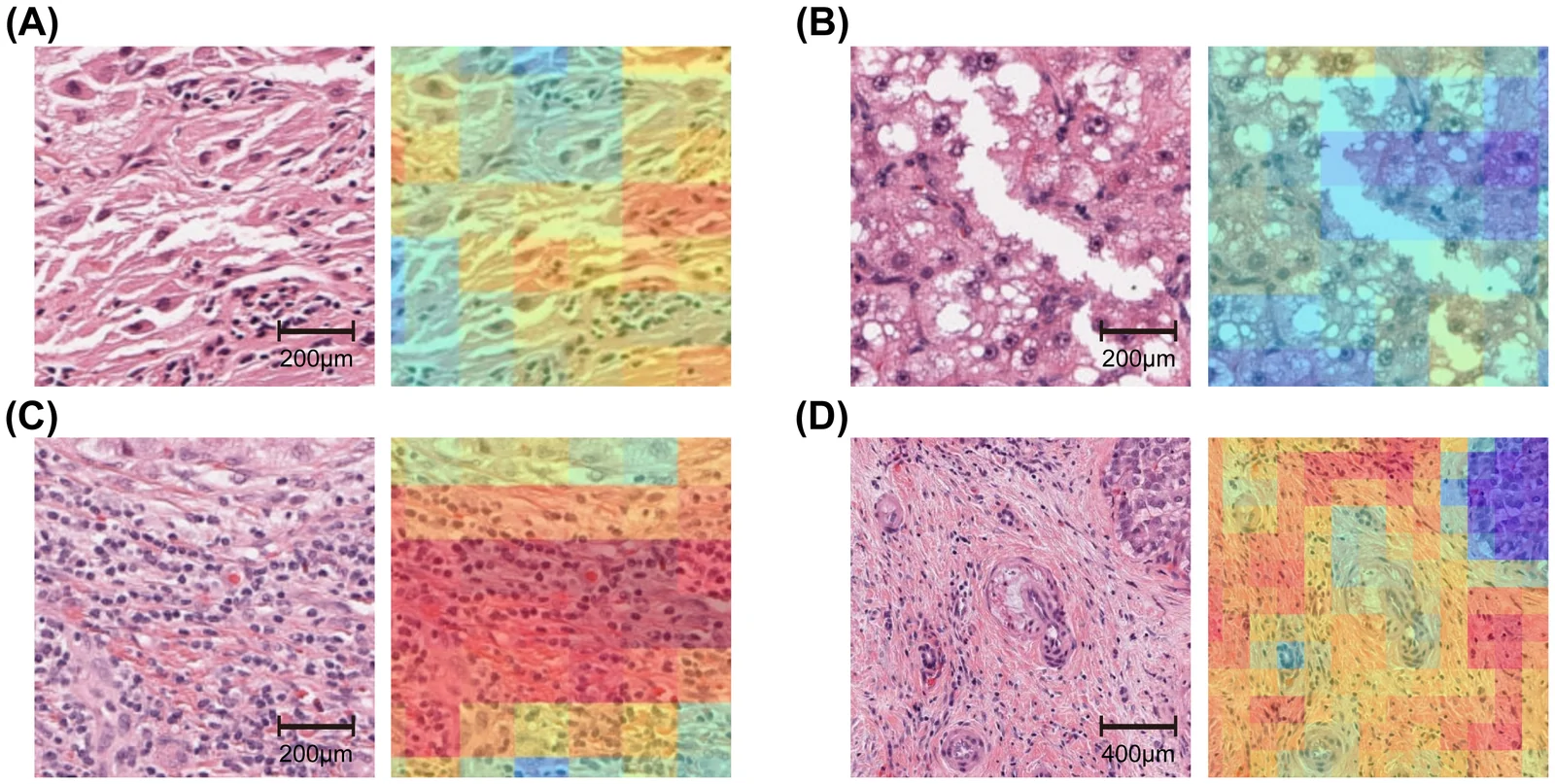
High-attention regions of representative WSIs and the corresponding heatmaps. **(A)** A poorly differentiated region and the corresponding heatmap. **(B)** A necrotic region and the corresponding heatmap. **(C)** An immune infiltration region and the corresponding heatmap. **(D)** A vascular invasion region and the corresponding heatmap.

### 3.2 Patient risk stratification

Risk stratification was conducted utilizing the Kolmogorov–Smirnov (K–S) curve to assess the model’s discriminative capacity. An elevated K–S statistic signifies enhanced differentiation between patients with varying outcomes at a specific risk-score threshold. As depicted in **Figure 4A**, the K–S statistic progressively increased with rising risk scores, peaking at 0.5380 at a risk score of 54.

**Figure 4:**
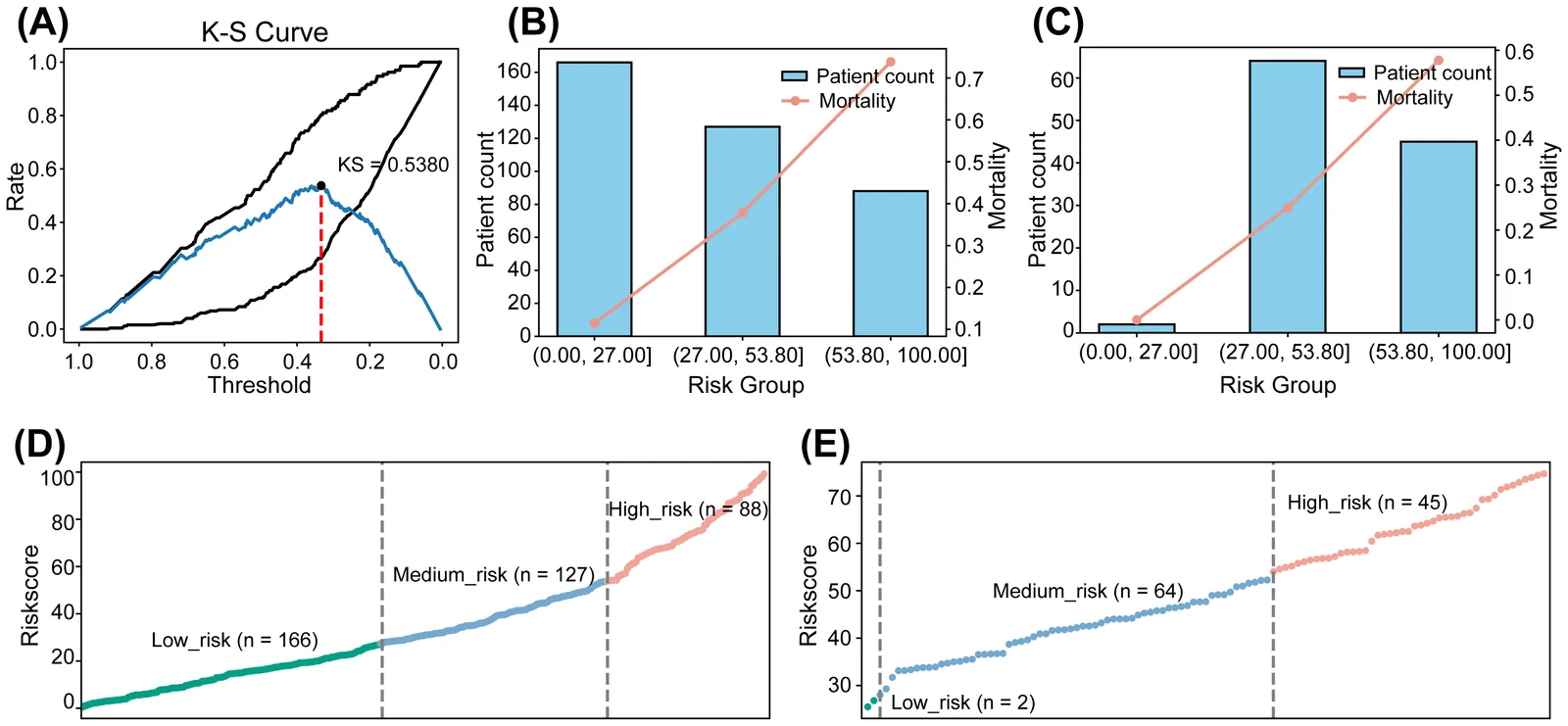
Kolmogorov–Smirnov (K–S) curve analysis and patient risk stratification. **(A)** K–S curve. **(B)** Mortality rates and patient counts across different risk groups in the TCGA cohort. **(C)** Mortality rates and patient counts across different risk groups in the institutional validation dataset. **(D)** Risk scores and patient counts across different risk groups in the TCGA cohort. **(E)** Risk scores and patient counts across different risk groups in the institutional validation dataset.

Based on the K–S curve analysis and the distribution of survival outcomes among patients with hepatocellular carcinoma, patients were categorized into three risk groups (**Figures 4B, C**):

- Low_risk: Scores ≤27
- Medium_risk: Scores >27 and ≤53.8
- High_risk: Scores >53.8

Mortality rates exhibited a progressive increase across the ascending risk groups in both the TCGA cohort (**Figure 4D**) and the institutional validation cohort (**Figure 4E**).

### 3.3 Prognostic comparison across risk strata

Survival analysis comparing the three risk groups revealed a consistent pattern across both cohorts. Patients classified as low_risk exhibited the most favorable survival outcomes, while those in the medium_risk group demonstrated moderate survival outcomes. Conversely, patients in the high_risk group experienced the poorest survival outcomes. This stepwise gradient in survival was statistically significant, as indicated by a log-rank test (P < 0.0001). **Figures 5A, B** illustrate the significant separation of the K–M survival curves in the TCGA and institutional cohorts, respectively.

**Figure 5:**
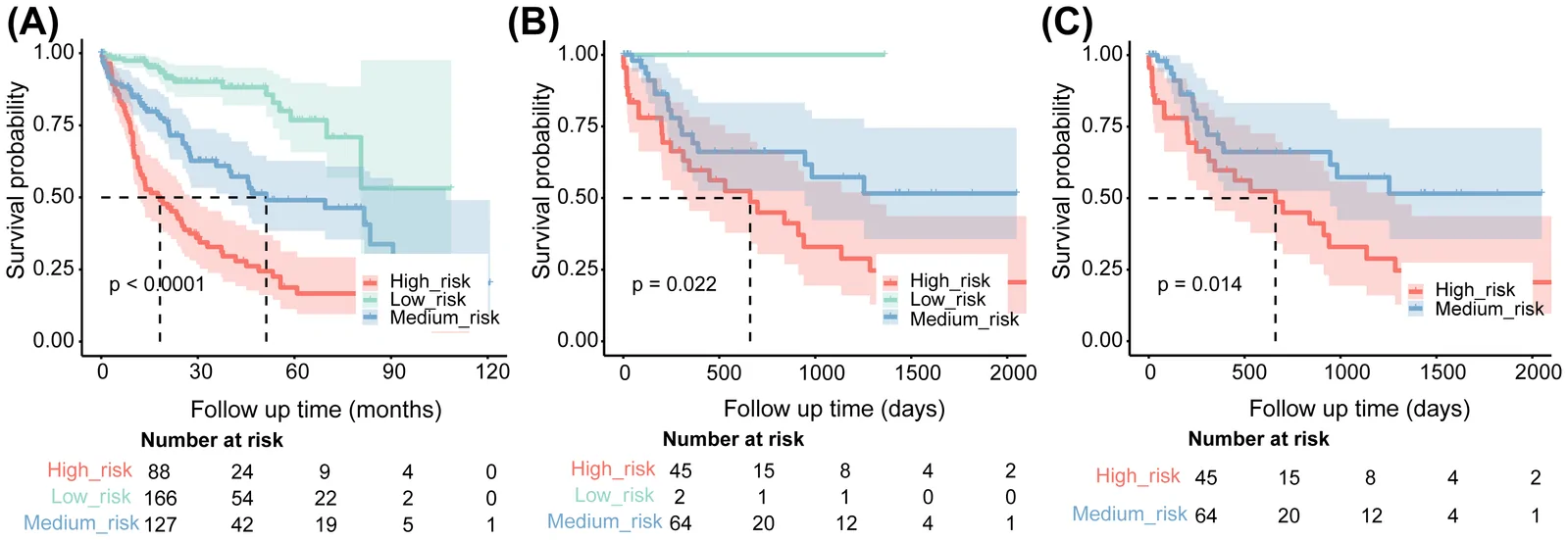
Survival analysis across different risk groups. **(A)** Survival analysis across different risk groups in the TCGA cohort. **(B)** Survival analysis across different risk groups in the institutional validation dataset. **(C)** Survival analysis across different risk groups in the institutional validation dataset after excluding the low_risk group. Due to the limited sample size, subgroup analyses within the external validation cohort are exploratory and intended to generate hypotheses.

Following the exclusion of the low_risk group due to its limited sample size, a *post hoc* sensitivity analysis was conducted (**Figure 5C**). The analysis revealed that significant survival differences remained between the medium_ and high_risk groups (P = 0.014). Given the relatively small sample size of the external validation cohort (n=111), these subgroup analyses should be considered exploratory and hypothesis-generating rather than definitive.

### 3.4 Comparison of treatment strategies across different risk groups

This analysis identified a total of 18 distinct treatment strategy combinations (**Figure 6A**). Among these, surgery alone, TACE alone, and the combination of surgery and TACE accounted for a substantial proportion of the treatment strategies (**Figure 6B**). We further compared the distributions of risk groups and survival status across the different treatment strategies (**Figure 6C**). Comparative analysis showed that patients in the high_risk group were more likely to receive immunotherapy, targeted therapy, and TACE than those in the medium_risk group. In contrast, surgery alone or surgery combined with other therapies was more frequently observed in the medium_risk group (**Figure 6D**).

**Figure 6:**
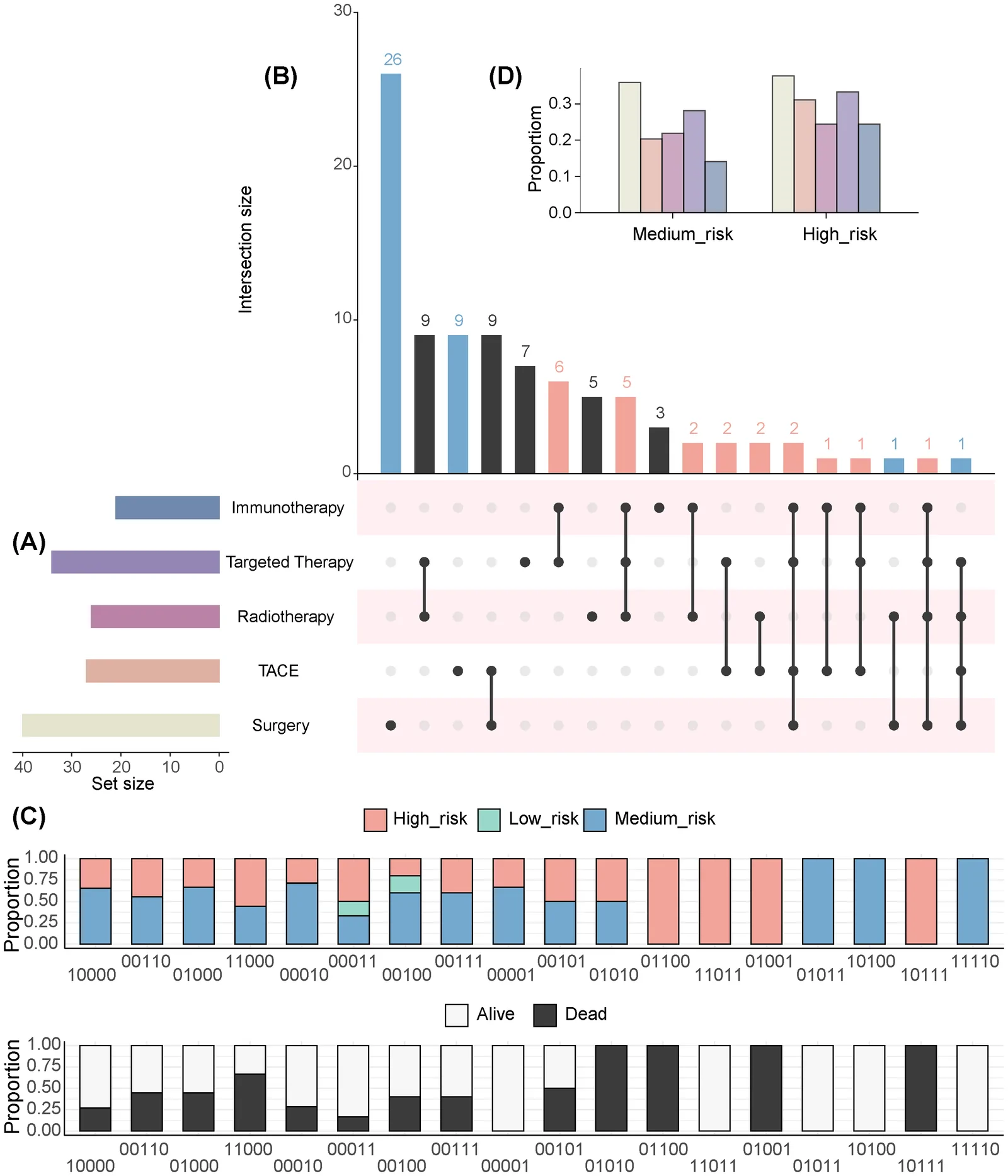
Comparison of treatment strategies across different risk groups. **(A)** Eighteen distinct treatment strategy combinations. **(B)** Intersection sizes of different treatment strategies. **(C)** Proportional distributions of different risk groups and patient prognostic status across the various treatment strategies. **(D)** Proportional distribution of treatment strategies across different risk groups.

We performed a comparative study to evaluate survival outcomes among patients who received the main treatment strategies versus those who underwent alternative treatments within each risk category. For the medium_risk group, opting for surgery alone, TACE alone, or surgery combined with other treatment methods was notably linked to better survival outcomes than other treatment approaches (P < 0.05; **Figure 7A**). In the high_risk group, strategies incorporating immunotherapy, targeted therapy, and TACE showed a tendency towards improved survival outcomes compared to other treatments; however, this difference did not reach the standard level of statistical significance (**Figure 7B**). Given the relatively small sample size of the external validation cohort (n=111), these subgroup analyses should be considered exploratory and hypothesis-generating rather than definitive.

**Figure 7:**
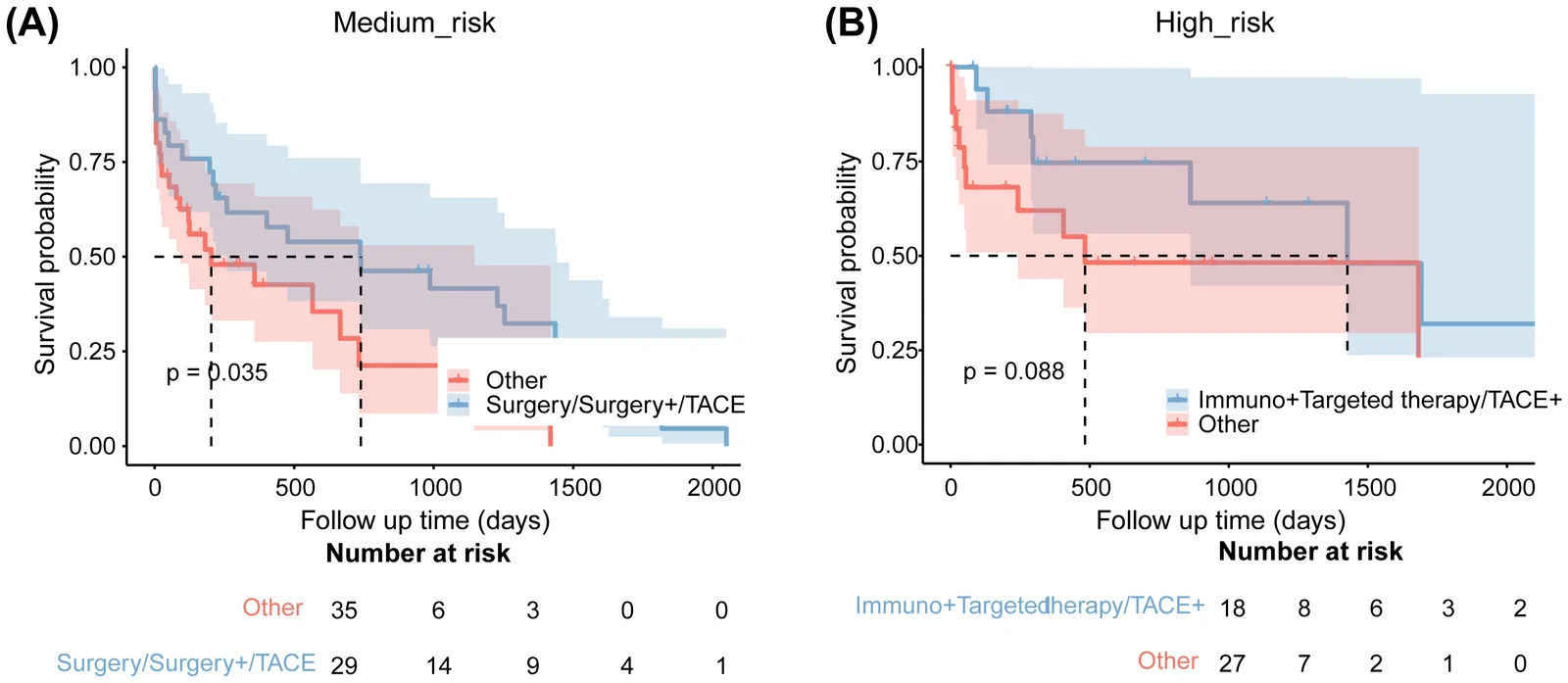
Survival differences within each risk group. **(A)** Survival differences associated with the predominant treatments versus other treatments in the medium_risk group. **(B)** Survival differences associated with the predominant treatments versus other treatments in the high_risk group. Due to the limited sample size, subgroup analyses within the external validation cohort are exploratory and intended to generate hypotheses.

These findings suggest that our risk stratification system has potential clinical utility for guiding treatment selection.

### 3.5 Construction of the NORMGRAM scoring system

To assess the potential of the proposed risk stratification system as an independent prognostic factor in hepatocellular carcinoma (HCC), a comprehensive analysis was conducted using univariate and multivariate Cox proportional hazards regression models, incorporating various clinical and pathological characteristics. Initially, variables such as T stage, N stage, M stage, AJCC stage, histological grade, sex, age, and risk group were examined through univariate Cox regression analysis. The analysis revealed that pathological T stage (pT stage), AJCC stage, and risk group were significantly correlated with survival (P < 0.05). Due to the overlap in prognostic information between pT stage and AJCC stage, only AJCC stage and risk group were included in the multivariate Cox regression model. Following adjustment by multivariable Cox proportional hazards regression for established confounders, including age, sex, tumor stage, AJCC stage, histological grade, and risk group, both AJCC stage and risk group were found to be independently associated with survival (P < 0.05). Forest plots derived from the TCGA cohort (**Figure 8A**) and the proprietary cohort (**Figure 8B**) consistently indicated that the risk group serves as an independent prognostic factor for HCC.

**Figure 8:**
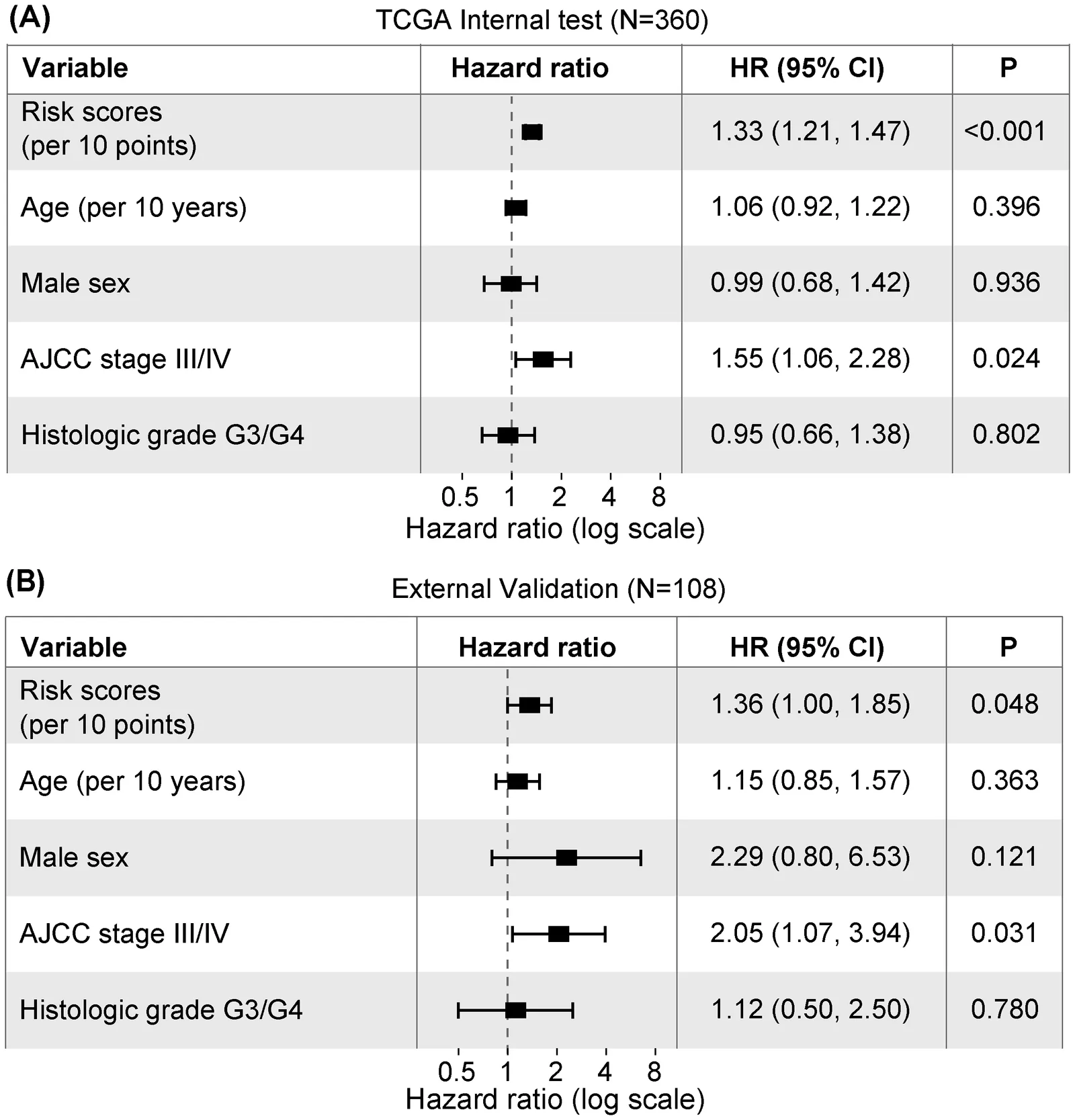
Forest plots. **(A)** Forest plot generated from the TCGA dataset. **(B)** Forest plot generated from the proprietary dataset.

To enhance clinical applicability, the prognostic variables identified through univariate and multivariate Cox regression analyses were incorporated into a nomogram using the rms package in R (**Figure 9A**). This nomogram was designed to predict overall survival (OS) probabilities at 1, 3, and 5 years. The time-dependent receiver operating characteristic analysis demonstrated that the nomogram achieved area under the curve (AUC) values of 0.838, 0.847, and 0.841 for predicting 1-, 3-, and 5-year OS, respectively (**Figure 9B**). Calibration curves indicated a strong concordance between the predicted and observed survival probabilities, reflecting satisfactory calibration of the nomogram (**Figure 9C**).

**Figure 9:**
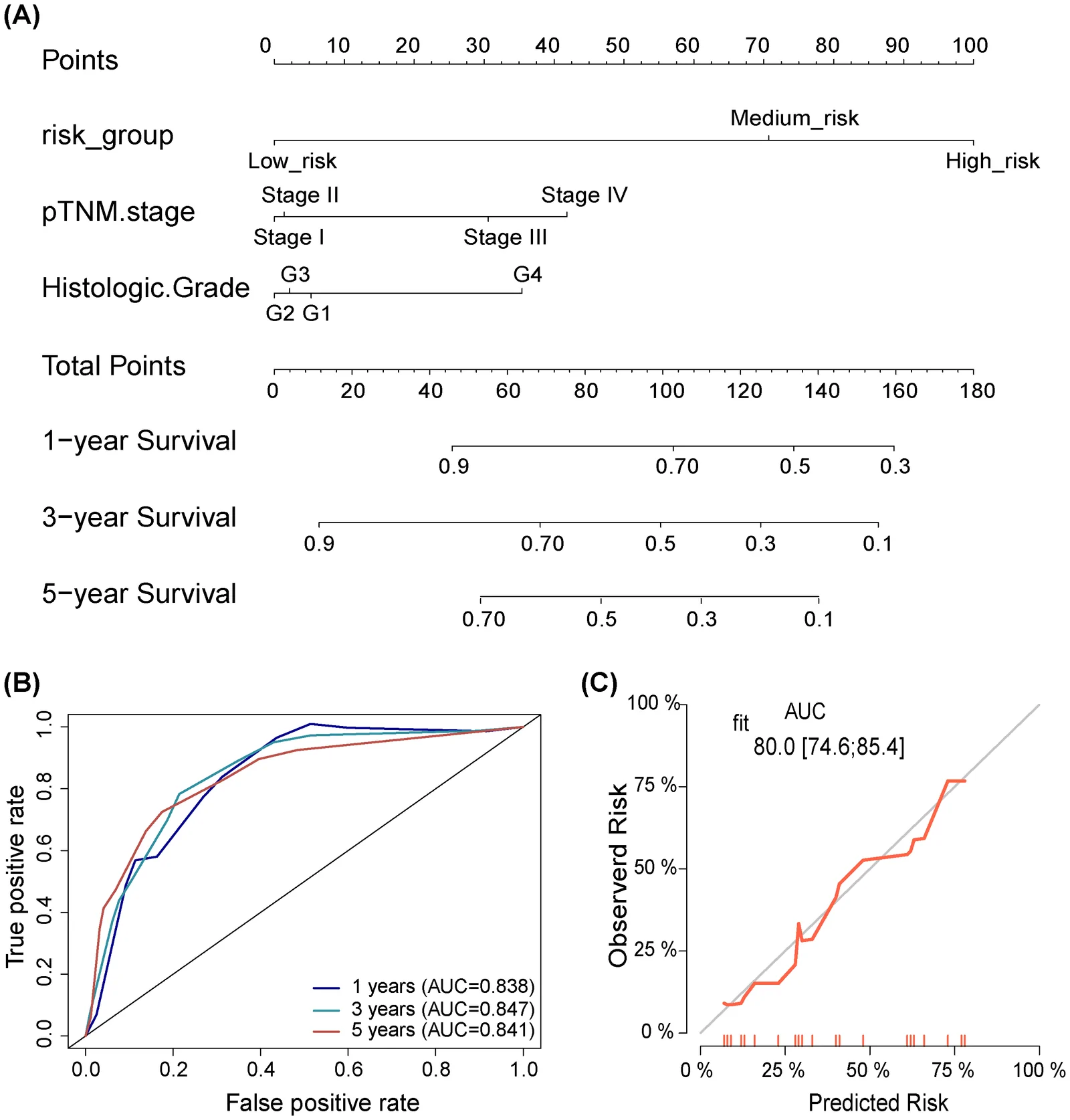
Nomogram construction and evaluation. **(A)** Nomogram constructed using the rms package in R. **(B)** The nomogram achieved AUC values of 0.838, 0.847, and 0.841 for predicting 1-, 3-, and 5-year OS, respectively. **(C)** Calibration curve analysis showing minimal deviations between the model-predicted probabilities and the observed outcomes.

Further analysis revealed a close alignment between the predicted risks and observed event frequencies across various risk groups (**Figure 10A**). Decision curve analysis (DCA) was conducted to assess the clinical utility of the nomogram. Compared to tumor stage, histological grade, or risk group alone, the nomogram, which integrates these prognostic factors, offered a greater net benefit over a wide range of threshold probabilities from 0.1 to 0.8 (**Figure 10B**), underscoring its potential clinical utility for prognostic assessment and decision-making.

**Figure 10:**
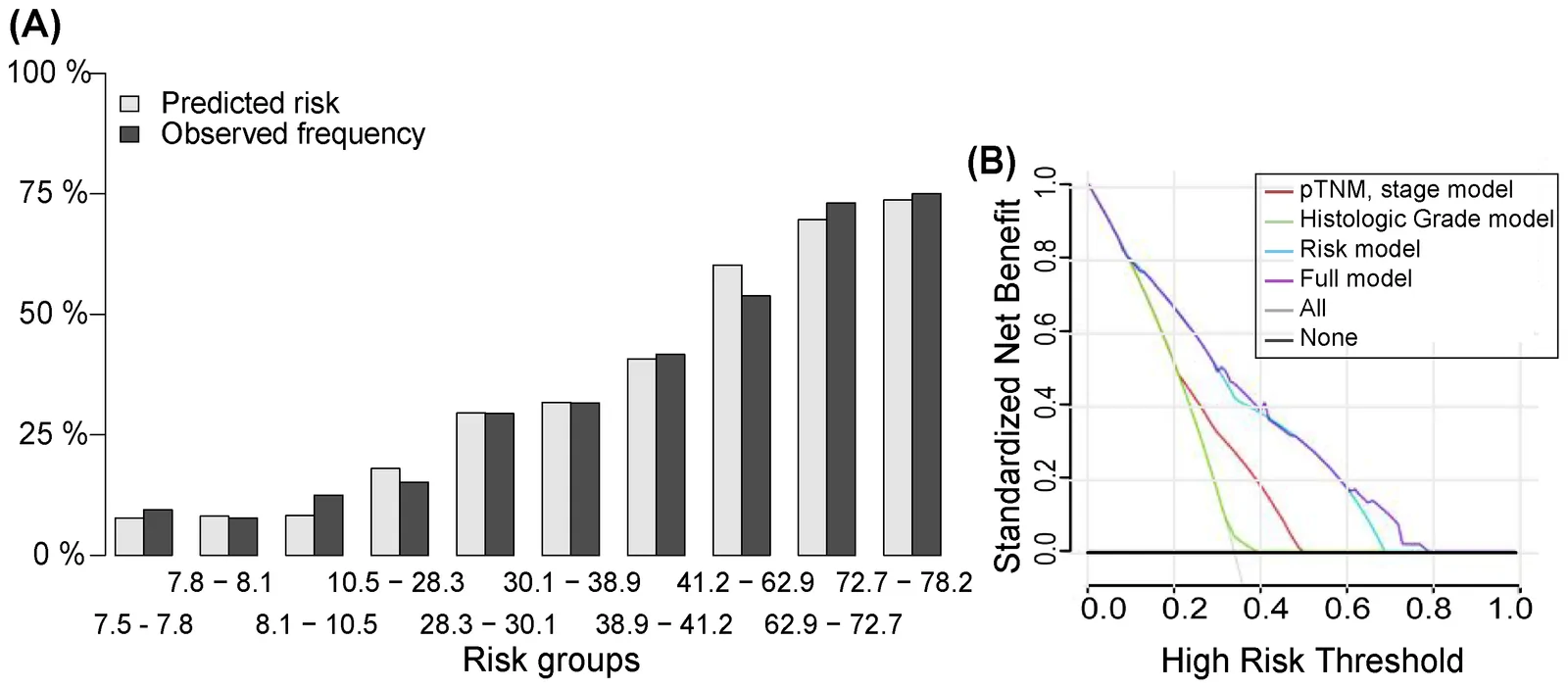
Further evaluation of the nomogram. **(A)** Predicted risk and observed frequency across different risk groups. **(B)** Decision curve analysis (DCA) comparing tumor stage, pathological grade, and risk group alone with the nomogram incorporating clinical features.

## 4 Discussion

In recent years, AI-powered pathomics has shown significant promise in various facets of hepatocellular carcinoma (HCC) management, encompassing diagnosis, treatment optimization, and prognostic evaluation. Previous research has developed prognostic models utilizing distinct patient cohorts. For instance, Shi JY et al. utilized a cohort comprising 1,125 HCC patients (2,451 whole-slide images, WSIs), along with an independent validation cohort of 320 HCC patients (320 WSIs) from The Cancer Genome Atlas (TCGA) (7). Similarly, Qu WF et al. constructed a model using data from 380 HCC patients who underwent liver transplantation (LT) to predict post-LT recurrence (6).

In contrast, this study utilized deep learning for prognostic risk stratification in patients with hepatocellular carcinoma (HCC) by employing two independent datasets: (1) the TCGA-LIHC cohort, which includes 365 HCC samples, and (2) an institutional cohort consisting of 111 HCC samples. The CLAM model was utilized for interpretable slide-level risk stratification. CLAM employs an attention mechanism to assess the contribution of individual histopathological patches to the final prediction (16). This high-throughput deep learning framework is specifically designed for weakly supervised classification tasks in computational pathology. Architecturally, CLAM integrates a trainable attention-based pooling function that consolidates patch-level features into discriminative slide-level representations, rendering it highly suitable for slide-level classification tasks (22). Consequently, our model was trained using CLAM’s attention-based multiple instance learning framework.

To control data leakage and ensure rigorous model validation, all data partitioning was performed at the patient level. All WSIs, patches, and features from the same patient were consistently assigned to the same training, validation, or test subset, with no sharing of patient-level information across subsets. For the TCGA-LIHC cohort, a patient-level StratifiedKFold approach was employed to construct 10 data partitions, each comprising training, validation, and internal test sets at a ratio of 7:1:2. Model parameters were updated using only the training set, whereas early stopping, model selection, and classification threshold determination were performed exclusively using the corresponding TCGA validation set. The internal test set was not involved in either model training or model selection.

For external validation, 20% of the external cohort was designated as an adaptation subset for model adjustment, whereas the remaining 80% was held out as an independent external test set. No patient-level information from the held-out external test set was used for model training, early stopping, hyperparameter selection, classification threshold determination, or probability calibration.

To address class imbalance during model training, a weighted BCEWithLogitsLoss function was used, with class weights calculated according to the distribution of deceased and surviving patients in each training set. The class weights were derived exclusively from the training data. Patient-level stratified sampling was used for data partitioning to maintain comparable class distributions across the training, validation, and internal test sets and to preserve, as closely as possible, the class distribution of the overall cohort.

Model performance was evaluated using multiple metrics, including ROC-AUC, accuracy, precision, recall, and F1-score, thereby avoiding reliance on accuracy alone, which can be affected by class imbalance. For external survival evaluation, the concordance index (C-index), time-dependent AUC, and bootstrap 95% Cis were additionally reported. No case duplication or cross-dataset oversampling was performed to address class imbalance during model training.

All subgroup analyses of treatment effects were performed using multivariable Cox proportional hazards regression with adjustment for potential confounders, including age, sex, TNM stage, AJCC stage, and histological grade. Adjusted HRs and corresponding P values are reported in the main text and **Figure 8**. Despite adjusting for various established clinical and pathological confounders in the multivariable Cox models, the possibility of unmeasured or residual confounding cannot be entirely ruled out.

The model outputs were primarily intended for patient risk ranking and discrimination rather than direct estimation of absolute mortality probabilities. Classification thresholds were determined exclusively using the TCGA validation set and were subsequently fixed for evaluation in both the internal test set and the external validation cohort.

The K-M survival analysis was conducted to assess the model’s efficacy in risk stratification. Variations in treatment patterns were identified among the risk groups, indicating that the proposed stratification may offer clinically pertinent insights for treatment selection (23). Notably, both univariate and multivariable Cox proportional hazards regression analyses, which included essential clinical and pathological characteristics, demonstrated that the risk group remained an independent predictor of survival in both the TCGA and institutional cohorts (24). Furthermore, decision curve analysis (DCA) revealed that the nomogram, which integrates risk group and histological grade, provided a superior net benefit over a wide range of threshold probabilities compared to models relying solely on individual clinical variables. Overall, these results underscore the potential clinical utility of the deep learning-based model for prognostic stratification of patients with HCC using histopathological WSIs and highlight its possible application in clinical decision-making (25).

In the K-M survival analysis, the low_risk group comprised a relatively small number of patients, potentially limiting the robustness of comparisons involving this subgroup. Consequently, a sensitivity analysis was conducted, excluding the low_risk group, to evaluate whether the survival difference between the medium_ and high_risk groups remained consistent. This limitation underscores the necessity of larger and more diverse datasets for robust model training and validation, as well as for enhancing generalizability across external populations.

The present study acknowledges several limitations. Firstly, due to the challenges associated with acquiring additional independent HCC whole-slide histopathology cohorts that include comprehensive long-term follow-up data, the external validation cohort was limited to 111 patients. This relatively small cohort size, coupled with the limited number of patients in certain subgroups following three-tier risk stratification, may have introduced statistical uncertainty and diminished the robustness of subgroup analyses. For the reasons mentioned above, these results should be considered exploratory and require validation in larger independent cohorts. Secondly, variations in hardware platforms, staining protocols, patient populations, and clinical follow-up practices across cohorts may have contributed to the observed decline in model performance within the external cohort. To further explore this issue, we conducted a comparison of feature distributions between the internal and external datasets to visualize inter-cohort feature drift following clinical matching. The findings indicated that differences in patient composition and follow-up design might partially account for the reduced performance in the external cohort. These analyses are detailed in the **Supplementary Materials**.

Future research should address this limitation by including larger and more diverse cohorts. Increasing both the size and diversity of the datasets could enhance the model’s generalizability across a wider range of patient populations and clinical settings. Although the current limitations exist, the model demonstrates potential clinical utility. However, further development and validation in larger, multicenter cohorts are necessary before clinical implementation.

## 5 Conclusion

This study developed and validated a prognostic model for HCC using histopathological WSIs from the TCGA and an institutional cohort. The model showed stable performance in internal and external validation. Kaplan–Meier survival analysis and Cox regression analyses showed that the proposed risk stratification was independently associated with patient prognosis and may help distinguish patients with different treatment patterns and survival outcomes. The nomogram integrating risk stratification with clinical variables further improved individualized survival prediction and may have potential value in clinical prognostic assessment.

## Data Availability

Publicly available datasets were analyzed in this study. This data can be found here: https://www.cancer.gov/ccg/research/genome-sequencing/tcga.
