# Supplementary material for: Leveraging deep learning for prognostic stratification of hepatocellular carcinoma using histopathological images
Source: Front Oncol. 2026 Sep 28;16:1849274. doi: 10.3389/fonc.2026.1849274 (PMC13655721; doi:10.3389/fonc.2026.1849274)
Supplement: Supplementary file 1 [file Table1.docx]

Supplementary material

S1. Feature distribution comparison experiments between internal and external datasets

| evidence | TCGA-LIHC | external dataset | Difference /statistic |
| --- | --- | --- | --- |
| Death rate | 35.1% | 37.8% | Overall – close |
| Median observation time | 601 days | 248 days | The external portion is noticeably shorter |
| Internal outcome recording during 90 days | 9.4% | 29.6% | +20.2 percentage points |
| Follow-up period: 1 year | 70.8% | 38.9% | −31.9 percentage points |
| Unmatch Alive/Dead AUC | 0.705（OOF） | 0.653（locked） | ΔAUC=−0.052 |
| After clinical 1:1 matching AUC | 0.679（OOF） | 0.653（locked） | ΔAUC=−0.026（95% CI −0.177～0.125） |
